# Sex-Specific Biochemical and Histopathological Effects of Chronic Meat-Based vs. Plant-Based Burger Consumption in a Rodent Model

**DOI:** 10.3390/foods14050888

**Published:** 2025-03-05

**Authors:** Cristina Filip, Ruxandra Ștefănescu, Bianca-Eugenia Ősz, Amalia Pușcaș, Corneliu Tanase, Adela Nechifor-Boilă, Amelia Tero-Vescan

**Affiliations:** 1 Department of Biochemistry, George Emil Palade University of Medicine, Pharmacy, Science and Technology of Târgu Mureș, 38th Gh. Marinescu Street, 540139 Târgu Mureş, Romania; cristina.filip@umfst.ro (C.F.); amalia.puscas@umfst.ro (A.P.); amelia.tero-vescan@umfst.ro (A.T.-V.); 2 Department of Pharmacognosy and Phytotherapy, George Emil Palade University of Medicine, Pharmacy, Science and Technology of Târgu Mureș, 38th Gh. Marinescu Street, 540139 Târgu Mureş, Romania; 3 Department of Pharmacology and Clinical Pharmacy, George Emil Palade University of Medicine, Pharmacy, Science and Technology of Târgu Mureș, 38th Gh. Marinescu Street, 540139 Târgu Mureş, Romania; bianca.osz@umfst.ro; 4 Department of Pharmaceutical Botany, Research Center of Medicinal and Aromatic Plants, George Emil Palade University of Medicine, Pharmacy, Science and Technology of Târgu Mureș, 38th Gh. Marinescu Street, 540139 Târgu Mureş, Romania; corneliu.tanase@umfst.ro; 5 Department of Histology, Center for Advanced Medical and Pharmaceutical Research, George Emil Palade University of Medicine, Pharmacy, Science and Technology of Târgu Mureș, 38th Gh. Marinescu Street, 540139 Târgu Mureş, Romania; adela.nechifor-boila@umfst.ro

**Keywords:** plant-based meat, processed food, biochemical markers, histopathology, metabolic health, liver function, lipid profile, leptin resistance, sex differences

## Abstract

The growing consumption of processed foods, including meat-based and plant-based burgers (PBM), raises concerns about their long-term health effects. While PBM burgers are marketed as healthier alternatives, their biochemical and histological impacts remain unclear. This study investigates the effects of chronic meat-based and PBM burger consumption on metabolic and organ health, considering sex differences. Thirty-six Wistar rats (18 males and 18 females) were divided into three groups: control (standard chow), meat burger-fed, and PBM burger-fed. Improved chow was prepared using lyophilized burger powder. Over eight weeks, food intake, weight gain, and food efficiency ratio (FER) were monitored. Serum biochemical markers, including AST, ALT, urea, creatinine, lipid profile (TG, CHOL, HDL, LDL), and leptin, were analyzed, alongside histopathological evaluation of the liver, kidneys, and heart. PBM-fed rats exhibited significantly higher FER than the meat group (*p* < 0.05). AST and ALT levels were slightly elevated in meat-fed rats, while PBM-fed males had increased urea levels. Triglyceride levels were significantly higher in the meat group, but cholesterol levels did not differ significantly. Serum leptin was elevated in both experimental groups, suggesting leptin resistance. Histological findings showed mild hepatic inflammation and microvacuolar changes, with minor cardiac fibrosis. These findings highlight the need for further research on PBM’s long-term health effects.

## 1. Introduction

The global shift toward sustainable protein sources is an urgent issue that requires international recognition. In recent years, plant-based meat (PBM) analogues designed to mimic conventional meat products have been extensively studied, particularly regarding consumer acceptance [1,2,3,4] and nutritional value [5,6]. However, concerns persist regarding the bioavailability and digestibility of their nutrients, primarily proteins [7]. Further investigation into their long-term health implications through in vivo studies on human subjects and experimental animal models is essential for a comprehensive understanding of these novel food products [8,9,10].

Processed meat products, which are typically derived from red meat, are classified as “ultra-processed foods” according to the NOVA food classification system due to their high additive content and extensive technological processing [11]. Similarly, PBM analogues are also categorized as ultra-processed foods, although they differ in chemical composition, containing lower protein levels, higher carbohydrate content, increased salt, and a greater number of additives [12,13]. These ingredients and processing techniques are employed to replicate the sensory characteristics of conventional meat. Given the established association between ultra-processed foods and a higher incidence of chronic non-communicable diseases, assessing the health impact of PBM alternatives is significant [14].

Despite international dietary guidelines advocating for reduced consumption of red and processed meats while promoting sustainable protein alternatives, global meat consumption continues to rise [15]. PBM analogues target a broad consumer demographic, including omnivores, by closely replicating the appearance and taste of conventional meat products. The development of these products extends beyond nutritional considerations, addressing critical issues such as environmental sustainability, animal welfare, and global food security. Consequently, a thorough evaluation of their nutritional composition and the potential benefits or risks associated with long-term consumption is imperative [16].

This experimental study aims to evaluate the influence and sex differences of the chronic consumption of meat burgers and their PBM burger alternative on health by quantifying biochemical parameters and assessing the histological changes in the main organs affected by the long-term consumption of processed foods.

## 2. Materials and Methods

### 2.1. Sample Selection

The burger was chosen as a food product in this study because it is one of the most popular foods among individuals of all ages due to its sensory properties and its widespread presence in the food market [17]. Beef and vegetable burgers (PBM) were purchased from the supermarket. In the case of PBM burgers, they were selected to imitate similar meat products; the name on the label included the term “burger”, and the protein content was close to that of a meat burger (minimum 15 g protein/100 g). The proteins in the case of the PBM burgers came from peas and grains, the lipids were of vegetable origin (sunflower oil and coconut oil), and the carbohydrates included both low-glycemic index sources (starch from peas and grains) and high-glycemic index sources (glucose syrup), along with salt and 8 food additives (methylcellulose, flavors, spices, ascorbic acid, citric acid, vinegar, concentrated beetroot juice, and malted barley extract). Based on the digestibility-corrected amino acid score (PDCAAS), the quality of pea proteins is quite similar to those of eggs (0.8–0.9 vs. 1.0 in the case of egg proteins); they are rich in lysine, an amino acid generally deficient in vegetable proteins, and the data in the literature recommend combining them with cereal proteins to obtain a PDCAAS score of 1.00 [18,19].

The beef burger label only specifies beef (98.9%), salt, and pepper. The caloric intake/100 g is similar between the PBM and meat burgers (231 vs. 238). The meat burger, compared to the PBM burger, had a higher content (g/100 g product) of proteins (19 vs. 16) and lipids (18 vs. 16) but lower salt (1 vs. 1.7). The nutritional composition of meat and PBM burgers compared to the standard diet is presented in Table 1. The caloric intake of the standard diet is much lower (only 87.45 kcal/100 g), being lower in lipids, lacking salt, and having a similar protein content to meat burgers.

### 2.2. Preparation of Improved Chow

**Figure 1:**
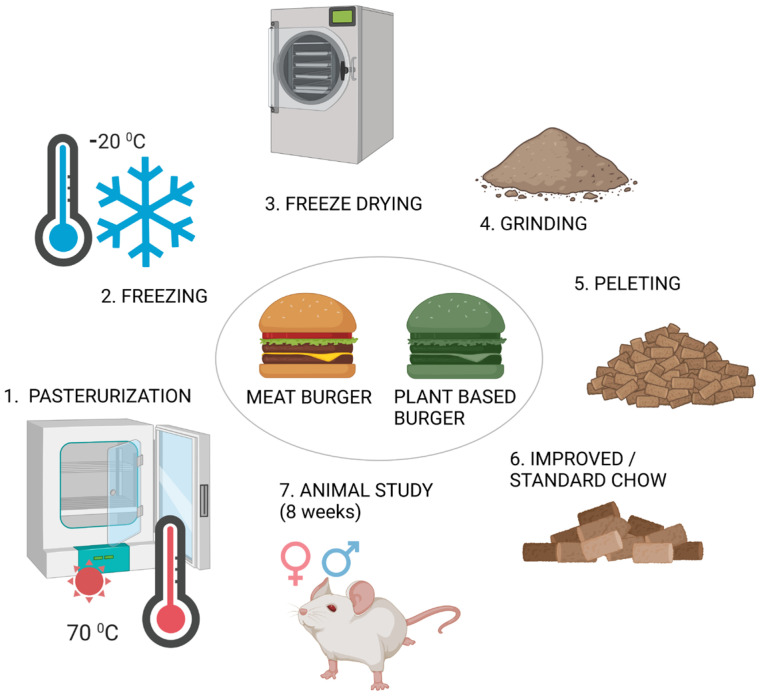
Schematic diagram of improved chow preparation (image created in BioRender (https://BioRender.com, accessed on 25 November 2024)).

Standard animal chow was purchased from the “Cantacuzino” National Institute for Medical and Military Research and Development in Bucharest. The burgers were kept in the freezer until processing. This involved vacuum sealing in plastic bags, pasteurization at a temperature of 70 °C within the product, and then freezing at −20 °C. The frozen burgers were lyophilized, ground into powder, homogenized with standard chow, and then pelleted. The animal chow was prepared similarly to other studies in the literature [20], with the steps being schematically presented in Figure 1.

The calculation of the amount of meat burger to be included in the improved chow administered was performed considering the average daily meat consumption in humans, with the global average being 17 g/day; however, there are very large variations, ranging from 30 g/day in Central Europe to 3 g/day in poorer countries in Asia and Africa [21]. Regarding daily red meat consumption, nutritional guidelines offer contradictory data, ranging from the 98 g/day recommended by the EAT Lancet Commission on Healthy Diets from Sustainable Food Systems to the 71 g/day recommended by the American Institute for Cancer Research, and the 85–100 g/week recommended by the Dietary Approaches to Stop Hypertension (DASH) [21]. To maintain a balanced nitrogen balance, the World Health Organization (WHO), the Food and Agriculture Organization of the United Nations (FAO), and the United Nations University (UNU) recommend a consumption of 0.8 g protein/kg bw/day [22]. For the extrapolation of the available data on human protein consumption in the case of rats, an allometric scaling factor was used.

Considering that rats of both sexes, weighing between 230 and 483 g, were used in this study, the average amount of standard chow consumed was 23 g. The improved chow contained 56.49% (*w*/*w*) of the fresh meat burger and 53.46% (*w*/*w*) of the fresh PBM burger. The improved chow’s composition was calculated based on the nutritional composition of the burgers and was similar to that in other studies (Table 2) [23].

### 2.3. Animals Included in This Study

This study included 36 animals, aged 36 weeks, that were randomly assigned to 3 groups as follows: the control group (C) (*n* = 12, 6 males—CM, and 6 females—CF), which was fed standard chow, the meat group (M) (*n* = 12, 6 males—MM, and 6 females—MF), which received improved chow enriched with meat burgers, and the PBM group (P) (*n* = 12, 6 males—PM, and 6 females—PF), which was fed improved chow enriched with PBM burgers. The Food and Drug Administration (FDA) recommends including an equal number of at least 5 females and 5 males in toxicity studies; however, to exclude the risk of mortality, one additional animal was added to each group [24]. The animals were obtained from the Animal Facility of George Emil Palade University of Medicine, Pharmacy, Science and Technology of Targu Mures, based on the Scientific Research Ethics Committee agreement (No. 2209/15.03.2023).

### 2.4. Animal Procedures

The animals were raised under standard laboratory conditions: an experimental temperature of 21–22 °C, a relative humidity of 40–60%, and a light period of 12 h. All experiments were performed in accordance with the European Communities Council Directive 2010/63/EU of 22 September regarding the protection of animals used for scientific purposes [25].

The total duration of the experiment was 8 weeks, preceded by 1 week of adaptation to the laboratory conditions, during which the subjects received standard feed, in accordance with other studies in the literature [23]. During the experiment, the animals received food and water ad libitum, a method that is considered stress-free for animals, which simulates human eating behavior as accurately as possible. The weight of the animals was monitored throughout the study via weekly weighing, and the amount of food consumed was measured. At the end of the 8 weeks, the animals were anesthetized via inhalation of isoflurane, and blood and biological tissue samples (liver, kidneys, heart, perivisceral adipose tissue) were collected for histopathological determinations. The animals died by exsanguination under general anesthesia.

### 2.5. Determination of Biochemical Parameters

The collected blood samples were centrifuged for 5 min at 3000 rpm using an 810 Eppendorf centrifuge, and the collected serum was stored at −80 °C until the day of analysis. The analysis of the biochemical parameters aspartate aminotransferase (AST), alanine aminotransferase (ALT), urea (UR), creatinine (CR), triglycerides (TG), total cholesterol (CHOL), HDL cholesterol (HDL), and LDL cholesterol (LDL) was performed using a Cobas Integra 400 Plus device with kits purchased from Roche Diagnostics (Mannheim, Germany). The analysis method and reagents used are described in Table 3. Serum leptin was determined by enzyme-linked immunosorbent assay (ELISA) using a kit for rat samples from EIAab SCIENCE INC, Wuhan, China, with a detection range of 0.312–20 ng/mL, according to the manufacturer’s recommendations.

### 2.6. Analysis of Biological Tissue Samples

After euthanizing the animals, liver, kidney, and heart tissue samples were taken. They were rinsed and preserved in 10% neutral buffered formalin (NBF). Tissue samples of the liver, heart, and kidney from six randomly selected rats (three female rats and three male rats) from each of the studied groups (18 cases in total) were sampled and further processed for histological examination. The histological processing involved dehydration, clearing, paraffin embedding, 5 mm sectioning, and staining with hematoxylin–eosin (HE) and Van Gieson staining (for some selected cases in which fibrosis was documented on HE staining). The analysis of the histological samples was performed using an Olympus BX51 microscope (Olympus life and material science Europa GMBH, Hamburg, Germany); bright field images were obtained using an Olympus SP350 digital camera and further processed using Olympus cellSens software (Version 3.1). Histological changes were assigned a severity score (0 = no changes, 1 = minimal, 2 = mild, 3 = moderate, and 4 = severe) according to the International Harmonization of Nomenclature and Diagnostic (INHAND) standards [26].

### 2.7. Statistical Analysis of the Results

The data were collected and stored using Microsoft Excel (Microsoft Office™) software (Office 365). The results were expressed as mean ± standard deviation (SD). Statistical analysis was performed using Graphpad PRISM 10 (Graphpad™). The results were analyzed using two-way repeated analysis of variance (ANOVA), with gender and diet as categorical variables. Further analysis was carried out using the Benjamini and Hochberg test as a correction for multiple comparisons. A value of *p* < 0.05 was considered statistically significant.

Statistical tests were not applied to the histopathological data; the results are expressed as the occurrence frequency of modifications/group.

## 3. Results

### 3.1. Food Intake and Weight Gain

The animals in the studied groups were weighed at the beginning of the experiment and again after 56 days from the beginning of the experiment, and they were kept in separate cages. At the end of each day, leftover food was removed and weighed to accurately calculate food intake. No significant differences were recorded regarding the amount of food consumed by the females and males of the three groups. The food efficiency ratio (FER) was calculated as the ratio of total weight gain (g)/total food intake (g/day). In the case of this last parameter, there were significant differences (*p* < 0.05) between the PM and MM groups, indicating better efficiency of food conversion into body mass. This could be explained by the higher carbohydrate content and the insulin-sensitizing effect of the plant-based burgers.

**Figure 2:**
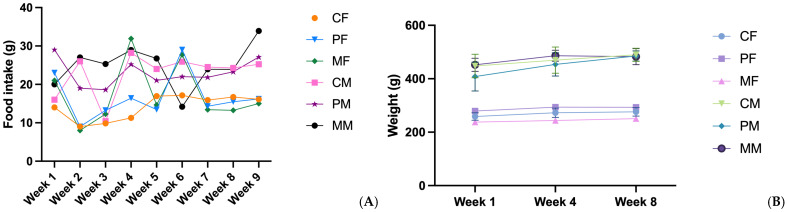
Schematic representation of weekly food consumption (**A**) and body weight (**B**) in rats.

The animals in the studied groups were weighed at the beginning and after 56 days of the experiment, and individuals were kept in separate cages. At the end of each day, leftover food was removed and weighed to accurately calculate food intake. No significant differences were recorded regarding the amount of food consumed by the females and males of the three groups. The food efficiency ratio was calculated as the ratio of total weight gain (g)/total food intake (g/day). In the case of this last parameter, there were significant differences (*p* < 0.05) between the PM and MM groups, indicating better efficiency of food conversion into body mass (Table 4, Figure 2).

### 3.2. Biochemical Parameters

#### 3.2.1. Aspartate Aminotransferase (AST) and Alanine Aminotransferase (ALT)

**Figure 3:**
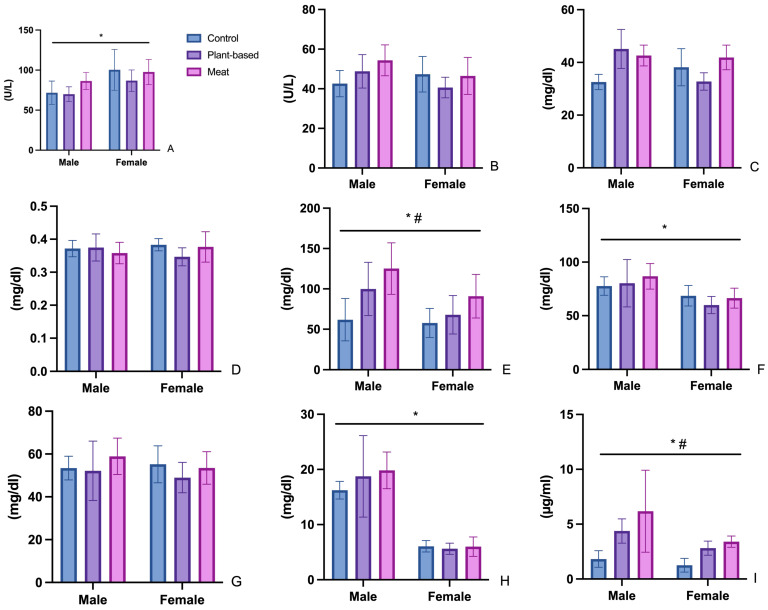
Graphical representation of (**A**) aspartate aminotransferase, (**B**) alanine aminotransferase, (**C**) urea, (**D**) creatinine, (**E**) triglycerides, (**F**) total cholesterol, (**G**) HDL—cholesterol, (**H**) LDL—cholesterol, and (**I**) leptin values after 60 days of feeding with standard diet or feed improved with meat or PBM burgers. *—indicates gender effect, #—indicates diet effect.

Diet did not significantly influence the AST and ALT levels. Significant gender differences were observed in the AST levels (F (1, 30) = 13, *p* < 0.0011). For AST, the highest values were found in the CF subgroup, followed by the meat groups, MF and MM. The highest values for ALT were observed in the meat-treated males subgroup (MM), followed by the subgroup of meat-treated females (MF). These findings suggest a potential trend of elevated ALT levels in meat-based subgroups, particularly among males. However, the lack of statistical significance implies that these variations might not be attributable to the dietary interventions (Figure 3).

#### 3.2.2. Urea (UR)

No gender or diet effects were observed in urea levels. There were, however, significant differences between PF and MF, but these values in the tested groups were not different compared with the control group. In the male group, the urea values in the treated groups (PM and MM) were higher compared with those in the male control group. The highest values for UR were observed in the PM subgroup, with mean values of 45.12 ± 7.38 mg/dL, followed by the meat group, with mean values of 41.90 ± 4.65 mg/dL for females (MF) and 42.63 ± 3.94 mg/dL for males (MM) (Figure 3).

#### 3.2.3. Creatinine (CR)

The female subgroup and the male subgroup presented similar values of CR, indicating no significant sex-based differences in this parameter. The highest values were observed in the meat female subgroup, MF. The values were very similar among groups, ranging from 0.39 to 0.43 mg/dL for all groups. This narrow range suggests that dietary interventions did not have a substantial impact on creatinine levels and that the observed variations are minimal (Figure 3).

#### 3.2.4. Triglycerides (TG), Total Cholesterol (CHOL), HDL Cholesterol (HDL), and LDL Cholesterol (LDL)

For TG, a significant main effect of diet was detected (F (2, 30), *p* = 0.0006), as well as gender differences (F (1, 30) = 6.7, *p* = 0.0147). TG levels were notably increased in the meat consumption group for both females and males. The difference between the females in the plant-based group and the females in the meat group is very high, with mean values of 125.20 mg/dL compared with 61.81 mg/dL (Figure 3).

For total cholesterol, the main effects on gender were observed (F (1, 30) = 15, *p* < 0.0005). For total cholesterol, the results did not indicate significant effects of diet. The highest total CHOL value was recorded in the meat-based male subgroup (MM). Despite this, all groups exhibited CHOL values comparable to the control group, indicating no substantial deviation from baseline levels. Among all subgroups, the plant-based female subgroup (PF) displayed the lowest total cholesterol levels (Figure 3).

HDL levels were consistent across all groups, with no statistically significant differences observed. LDL levels, on the other hand, exhibited notable variations between females and males (F (1, 30) = 112.3, *p* < 0.0001). However, these differences in LDL levels were not statistically significant when comparing the treated groups (meat-based and plant-based) to the control group, suggesting that the type of diet did not significantly impact LDL levels (Figure 3).

### 3.3. Leptin

Regarding leptin levels, significant gender (F (1, 30) = 8.591, *p* = 0.0064) and diet (F (2, 30)–11.59, *p* = 0.0002) effects were noticed. However, there was no significant interaction between gender and diet (F (2, 30) = 1.311, *p* = 0.2846). Among the studied groups, the lowest serum leptin values were found in the CF group, and the highest were found in the MM group. The Free Leptin Index was calculated for the studied groups as the ratio of the serum value of leptin (ng/mL) to body mass (g) (Figure 3, Table 5).

### 3.4. Histopathological Analysis

Three rats were randomly selected from each of the studied subgroups of six animals for histopathological analysis. The histological changes observed in the analyzed tissue are presented in Table 6.

**Figure 4:**
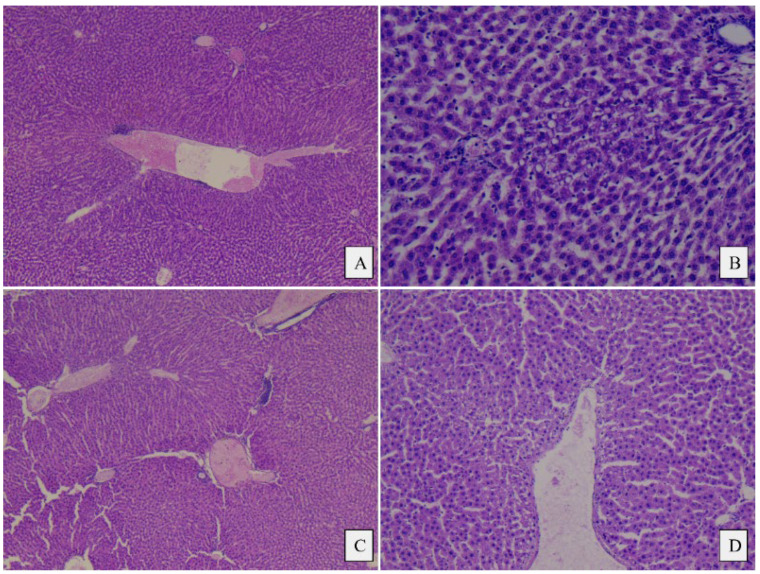
Histopathological images of the liver from the control and plant-based groups: (**A**) Control, male, minimal area of centrilobular inflammatory infiltrate, 5×; (**B**) Control, female, small area of microvacuolar fatty dystrophy, 20×; (**C**) Plant-based group, male, minimal area of intraparenchymal inflammatory infiltrate, 5×; (**D**) Plant-based group, male, microvacuolar dystrophy, 10×.

**Figure 5:**
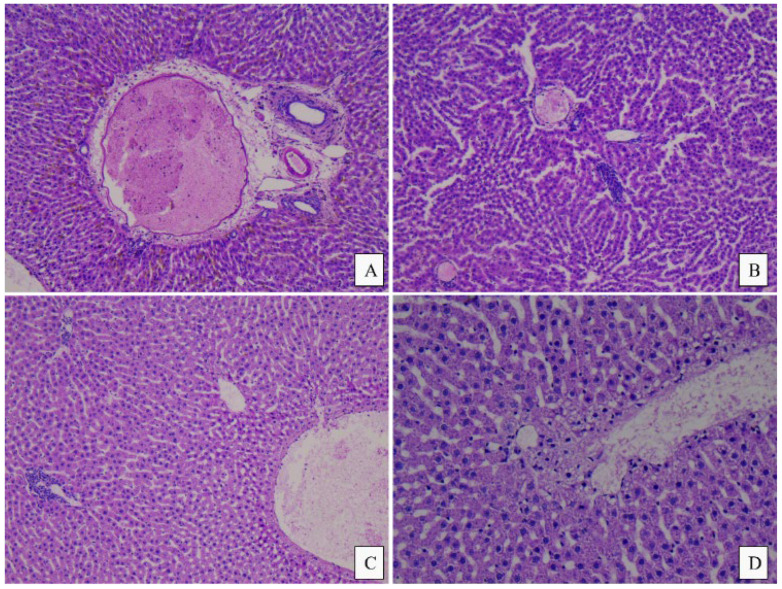
Histopathological aspects of the liver in the meat group: (**A**) Female, normal-appearing port space with rare plasma cells at this level, 10×; (**B**) Female, intraparenchymal inflammatory infiltrate, 10×; (**C**) Male, minimal periportal inflammatory infiltrate and microvacuolar fatty dystrophy, 10×; (**D**) Male, microvacuolar fatty dystrophy, 20×.

**Figure 6:**
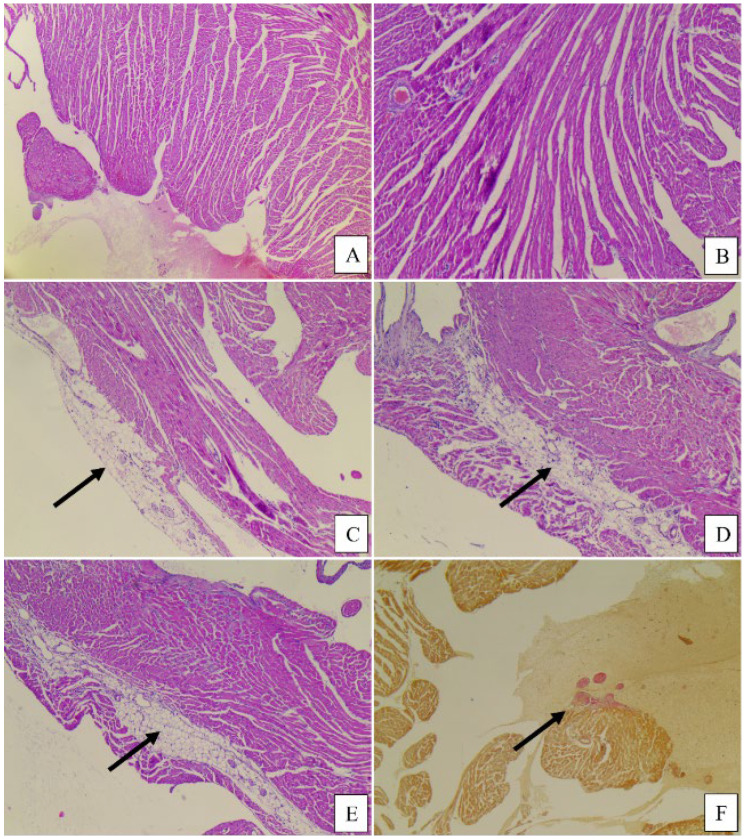
Cord histopathological aspects: control, plant-based, and meat groups, hematoxylin–eosin staining: (**A**,**B**) Control, male, myocardial tissue of normal histological appearance, 5× (**A**), 10× (**B**); (**C**,**D**) Plant-based, 2 cases, with subepicardial adipose tissue (**C**) and intramyocardial adipose tissue (arrow), (**D**) 5×; (**E**,**F**) Meat, 2 cases, intramyocardial adipose tissue, 5× (**E**), and minimal area of subendocardial fibrosis highlighted in van Gieson stain, 5× (**F**).

No significant morphological differences were observed among the three study groups. The heart, liver, and kidney tissue samples generally revealed a normal histological appearance, with some slight variations as follows: In the control group and the meat group, the liver showed minimal areas of microvacuolar dystrophy, whereas in the PBM group, the liver had a normal appearance with no modifications (Figure 4 and Figure 5). The cord revealed a minimal area of subendocardial fibrosis in the MM, VF, and VC subgroups. The VM and CM subgroups exhibited a minimal/moderate area of subendocardial fibrosis, as well as minimal lymphocytic inflammatory infiltrate (Figure 6). The kidney revealed a normal histological appearance in all study groups (Table 6).

## 4. Discussions

### 4.1. Food Intake and Weight Gain

Body weight was measured every week (Table 5), and it was observed that all groups in this study experienced an increase in body weight, with male rats having a higher body weight than female rats. The greatest weight gain occurred in males from the PM group, but without significant differences compared to the MM and CM groups (51.66 g compared with 30.33 g and 42.00 g, respectively). Only FER values differed significantly in the PM group compared to the MM group, which could be explained by the higher carbohydrate content and the insulin-sensitizing effect of the plant-based burgers.

There were no significant differences in the weight gain of groups CF (17.17 g), PF (13.67 g), and MF (13.17 g). This increase in body weight is proportional to the amount of food intake in each group. Our results are similar to research from 2024 that studied the effect of plant-based meat on lipid metabolism [9] and showed that mice fed plant-based pork and beef gain weight faster and more significantly compared with those in the meat groups. Xie et al. also affirm that weight gain is not influenced strictly by caloric intake but also by activity level. Plant-based meat products, compared with meat products, contain more additives [27]; for example, in our case, the beef burger contains only meat, and the plant-based version contains several additives for specific technological purposes and sensory qualities. The use of additives can improve the taste of food, making it more pleasant and increasing appetite.

Although the nutritional content of the two types of burgers is apparently balanced, with no major differences in caloric, protein, lipid, carbohydrate, or salt content, differences between the diets are still observed when calculating the dietary inflammatory index (DII) based on data provided by Shivappa et al. (2014) [28]. The DII score considers the pro-inflammatory and anti-inflammatory properties of 45 food ingredients, including their caloric, protein, carbohydrate, and lipid content, as well as vitamins and minerals [29].

Having only a few general data points about the composition of proteins, carbohydrates, or lipids on the product label, an attempt was made to calculate the DII based on these values to obtain a general framework of the pro/anti-inflammatory potential of these foods and to possibly correlate it with the results obtained. The data obtained show a DII score for meat burgers of −0.85, for PBM of −0.76, while the normal diet has a score of −1.46. These values show anti-inflammatory potential for all three types of diets, with higher anti-inflammatory potential values for the standard diet + 23% lyophilized meat burger compared to the standard diet + 23% lyophilized PBM burger. These values are based on the higher protein content of the meat burger compared to PBM (21.45 g vs. 17.54 g), with proteins having a negative DII anti-inflammatory score (−0.25).

Regarding fat content, the product label mentions the total fat content without distinguishing between saturated and unsaturated fats. Fats have a positive DII score (+0.298); therefore, due to the fat content of the meat burger compared to the PBM (8.47 g vs. 4.83 g), both foods receive a positive score, even if the content of (poli)unsaturated fats like omega-3 or -6 fatty acids would result in a negative score, which would be obvious in the case of the PBM. Carbohydrates have a slightly negative DII score (−0.1), and the carbohydrate content of the PBM is significantly higher than that of the meat burger (4.83 g vs. 0.4 g). Again, in the case of the PBM burger, the total carbohydrate content is considered without distinguishing between the types of carbohydrates, with it being known that dietary fiber presents a strongly negative DII score (−0.663). The literature data show that a veggie burger has an average fiber content of 5 g/serving, which would significantly modify the total DII value of the PBM burger by approximately (5 g × −0.663). The salt content of the two types of burgers is elevated, being higher in the case of the PBM (0.39 g vs. 0.03 g), and contributes to a pro-inflammatory DII (+0.5). The caloric intake of the meat burger compared to the PBM is similar and does not directly influence the DII calculation but serves as a reference for standardizing nutrient intake.

### 4.2. Analysis of Changes in Biochemical Parameters

#### 4.2.1. Aspartate Aminotransferase (AST) and Alanine Aminotransferase (ALT) Changes

AST and ALT are markers of hepatocellular injury with a very well-defined metabolic role. AST has a bilocular localization, with 60% in the cytoplasm and 40% in the mitochondria, while ALT is found only in the cytoplasm, specifically in hepatocytes at the periphery of the liver lobe. Serum transaminase values are physiologically higher in males than in females; those of ALT change much more significantly in liver diseases marked by cytolysis, while the values of both transaminases change in obese individuals [30]. AST and ALT values are increased in the group that consumed meat products (MM and MF) compared to the group that consumed plant-based products (PM and PF). The data obtained in our study are consistent with the literature, which shows that a diet rich in dietary fiber reduces AST and ALT levels, as well as the risk of non-alcoholic fatty liver disease (NAFLD) [31]. The literature defines the Healthful Plant-Based Diet Index (hPDI), which expresses an individual’s adherence to a diet rich in fiber from whole grains, vegetables, and nuts and is directly associated with a reduction in the risk of cardiovascular disease, diabetes, and overall mortality [32,33]. The label of the plant-based burgers used in our study states that they are a rich source of dietary fiber, even though their content is not expressed quantitatively.

On the other hand, ALT also plays an important role in amino acid metabolism, with variations in plasma levels serving as an indicator of adaptive mechanisms triggered to metabolize a greater number of amino acids [34].

#### 4.2.2. Urea (UR) and Creatinine (CR)

A diet based on vegetable proteins, due to its high fiber content relative to protein, significantly reduces nitrogenous compounds resulting from metabolism, such as urea, as well as uremic toxins (free and total; indoxyl sulfate and p-cresyl sulfate) [35]. A study by Xu et al. (2020) comparing serum parameters relevant for establishing renal function, including urea and serum creatinine, in 269 vegetarians and 269 omnivores shows that a vegetarian diet, due to its dietary fiber content, has a protective effect on renal function [36].

In the case of serum urea, a notable difference was observed in the PF female group compared to the MF group, as well as in the male rats in the PM and MM groups compared to the CM group. Data from the literature confirm sex differences in serum urea values, with serum values being higher in males regardless of diet type [37,38]. The CARDIVEG study, conducted on 107 subjects (82 women, 25 men; median age 52) with low or medium cardiovascular risk, shows that adopting a vegetarian diet for 3 months significantly reduced cardiovascular risk as well as renal function parameters, such as serum creatinine (−5.3%; *p* < 0.001), urea (−8.7%; *p* = 0.001), and the urea/creatinine ratio (−5.8%; *p* < 0.001), compared to a Mediterranean diet [39].

The serum creatinine values in all animals included in this study were within the normal range for rats (0.2–0.8 mg/dL in males and 0.2–0.7 mg/dL in females), with no significant effects induced by diet or gender [40]. Serum creatinine can be a result of the degradation of muscle creatine or from the consumption of animal protein. Since the three diet types contain similar amounts of protein, variations in serum creatinine levels are not influenced by diet type.

#### 4.2.3. Triglycerides (TG), Total Cholesterol (CHOL), HDL Cholesterol (HDL), and LDL Cholesterol (LDL)

In the case of TG, gender and diet differences were observed. The serum TG values in the male groups are higher than those in the female groups, with the highest values occurring in the MM group. A study conducted by Barros et al. in healthy adults of both sexes shows that serum TG values increase significantly post-burger consumption, regardless of its type, beef or PBM, but the increase in TG is much faster in the case of PBM [41]. PBM burgers usually contain, in addition to protein, other food additives to obtain the desired sensory properties, especially TG and carbohydrates. Even though there are data in the literature regarding the composition of different types of PBM burgers, data regarding their digestibility are still inconclusive and even contradictory [20]. In vitro digestibility studies show that lipolysis is increased in the case of beef burgers compared to PBM burgers (91.46 vs. 49.72 mg free fatty acids/g sample), which is attributed to the dietary fiber content in the PBM burger [1]. Other studies show the opposite: a much lower digestibility of beef burgers due to the dense matrix structure that prevents lipolytic enzymes from accessing TG [42]. Regarding sex differences in TG values, these can be attributed to estrogens that activate lipoprotein lipase in adipose tissue and muscle and favor the capture of TG in these tissues and decrease plasma values. They can also exert an insulin-sensitizing effect that favors the use of glucose in cells and prevents its transformation into TG.

Regarding serum CHOL values, they are less influenced by dietary intake, as serum CHOL originates from endogenous synthesis. The key enzyme in CHOL synthesis is β-hydroxy β-methylglutaryl-coenzyme A (HMG-CoA) reductase, which is activated by insulin released after dietary carbohydrate intake. For this reason, there are no major differences in CHOL values among groups of the same sex. Differences, however, appear between the blood lipoprotein fractions that contain CHOL, namely HDL and LDL, one of which has atherogenic potential and the other being antiatherogenic [43]. In women, LDL values differ depending on estrogen levels; before menopause, LDL values are lower than in men because estrogens stimulate the expression of hepatocyte LDL receptors, decreasing LDL in the blood and favoring reverse cholesterol transport to the liver (HDL). After menopause, LDL levels increase significantly, and studies show that LDL levels are much more difficult to manage than in men [44].

### 4.3. Leptin and Appetite Regulation

Leptin is a hormone secreted by adipose tissue in proportion to its quantity that is responsible for a plethora of metabolic effects, including the regulation of appetite, body mass, and lipolysis in adipose tissue via negative feedback in the hypothalamus, and presents systemic pro-inflammatory effects [45]. Although the mechanisms that protect the body against weight loss are well known (fibroblast growth factor 21 (FGF21), growth differentiation factor 15 (GDF15), liver-expressed antimicrobial peptide 2 (LEAP2), adiponectin, glucagon-like peptide-1 (GLP-1), and gastric inhibitory polypeptide (GIP)), the exact mechanisms that are triggered in response to overfeeding are still unknown [46].

The carbohydrate caloric intake of meat products differs significantly from that of vegetable products (0.1 g/100 g compared to 5.8 g/100 g), which is attributed to the need to add different types of starch to achieve the desired consistency and composition. Studies show that plant products have a higher degree of satiety compared to meat analogues [47]. Even considering that the total amount of food consumed/day was not different between animals of the same sex in the studied groups, an increase in weight was observed in the PM group.

Serum leptin values are increased in both females and males in groups that consumed processed meat or vegetal products. The phenomenon of leptin resistance caused by hyperleptinemia is well known and described in the literature and leads to a reduction in the expression of leptin receptors in the brain and, consequently, a decrease in its signaling mechanisms [48]. Calculating the Free Leptin Index shows the tendency of the animals to develop leptin resistance [49]. In the case of our study, although the greatest weight gain was observed in the PM group, this does not correlate with leptinemia values. In rats, the leptinemia values described in the literature vary between 0.5 and 5 ng/mL, with the standard weight of rats being between 200 and 400 g; the values considered within the normal limits of the leptin index are 0.003–0.01 ng/mL/g [50]. The leptin index values in the PF and PM groups are significantly increased (*p* < 0.05) compared to the control group (CM and CF), but they fall within the limits considered normal for this parameter. In the case of the MM and MF groups, the leptin index values are also significantly increased compared to the control group; however, the values are at the upper limit (MF) or exceed the normal limits (MM).

Leptin has a dual nature as a hormone and a cytokine, making the connection between the endocrine system, diet, and immune system and playing a role in the regulation of inflammatory processes [51]. The higher values of serum leptin in the plant (PM and PF) and meat (MM and MF) groups can also be attributed to the chronic high lipid content in the diet, which is a result similar to that obtained by El-Haschimi et al. in a study of mice fed a high-fat diet for 15 weeks [52].

Similar to our findings, the study conducted by Crimarco et al. on humans who received either PBM or a red meat diet for 8 weeks did not identify significant differences regarding serum values of several parameters of inflammation (IL-6, IL-18, TNF, IL-12B, IL-10, TGF-β) [10]. Animal studies, although they present the impediment of interindividual differences and interspecies extrapolation, have the advantage of correlating serum values with post hoc histological analysis. Although the leptin index did not show significant differences between the studied groups and the control, there was a tendency toward leptin resistance, which was confirmed by the initial significant modification of serum TG values that was probably followed by a subsequent decrease in HDL and an increase in LDL [53].

### 4.4. Histopathological Analysis

There are no apparent notable lesions in any organs isolated from rats in all three groups after 60 days of a modified diet. Our results indicate that there are no adverse toxic effects in rats fed plant-based analogues compared with those fed meat. The histopathology analysis of the liver indicates, in the case of the PM group, a minimal area of intraparenchymal inflammatory infiltrate and microvacuolar dystrophy, while in the MM group, a minimal periportal inflammatory infiltrate and microvacuolar fatty dystrophy is present. No notable changes appeared in the PF group, while in the MF group, only intraparenchymal inflammatory infiltrate was present. These results are consistent with other studies in the literature, as no significant pathological lesions in the liver were identified in mice of similar age and nutrition. The study by Xie Y et al. identified, in the plant-based groups, hepatocyte edema, cytoplasmic vacuolation, and punctate lymphocyte infiltration [9].

Although minimal histological alterations were observed in the livers of all animal groups fed a modified diet, these changes could have different underlying causes. Previous studies have demonstrated that a fast food diet administered to rats leads to non-alcoholic steatohepatitis [54].

The histological alterations observed in the groups of animals consuming the PBM diet might represent the liver’s response to a diet that is deficient in certain compounds. In addition to dietary fiber that can interfere with amino acid absorption, low vitamin B12 status and high levels of homocysteine after 8 weeks of study could be responsible for modifying the regenerative and reparative capacity of the liver [55,56]. On the other hand, choline deficiency can impair liver plasticity, resulting in NAFLD and muscle damage, which is characterized by modifications in serum values of liver enzymes and creatine kinase [57].

As mentioned previously, although we lack precise details about the fat content differences between the meat burger and the PBM, the declared fat amount in the meat burger is 1.8 times higher. In studies involving rats fed an essential fatty acid (EFA)-deficient diet for 9–12 weeks, signs of fatty liver were observed [58].

When an animal is exposed to a diet unsuited to its species, it is common to see inflammatory signs in the liver. However, over time, chronic inflammation can lead to liver fibrosis, cirrhosis, or even liver cancer. Thus, the progression from a “reparative” inflammatory response to cancer depends on the duration of exposure to the causative factor [59]—in this case, an inadequate diet.

On the other hand, previous studies have shown that plant-based burgers contain methionine at levels 21 times lower than those found in meat burgers [58], and a methionine deficiency in the diet predisposes individuals to fatty liver disease.

Dietary intervention plays a crucial role in the management of NAFLD, and literature studies indicate that the first line of intervention is either a hypocaloric diet or the Mediterranean diet, which is characterized by a high intake of vegetables and unsaturated fats from fish [60]. However, these findings are based on the use of a healthy vegetable-based diet and not on the use of PBM, which is considered fast food [61]. Although the literature reports sex differences in the prevalence of NAFLD due to hormones, obesity, and the gut microbiome, with premenopausal women being less likely to develop NAFLD, nutritional interventions must be personalized, and the gap in knowledge about sex differences in liver disease requires future studies.

The cord in the case of the PBM group presented subepicardial adipose tissue and intramyocardial adipose tissue, and the meat group exhibited intramyocardial adipose tissue and a minimal area of subendocardial fibrosis.

## 5. Conclusions

Chronic consumption of both meat-based and plant-based burgers leads to metabolic and histological alterations, with notable sex-specific differences, particularly in lipid metabolism, liver function, and leptin levels; while PBM burgers may offer certain advantages, their long-term health effects warrant further investigation to assess their overall safety and nutritional impact. Although one limitation of this study is the lack of mechanistic investigations, particularly regarding the pathways underlying the observed metabolic and histological changes, our primary objective was to identify the main organs affected and the key biochemical parameters that differ between sexes in response to the consumption of meat-based and PBM burgers. Future research should explore deeper mechanistic insights, including molecular and cellular analyses, to better understand the physiological responses to the chronic consumption of PBM and meat-based burgers.

## Figures and Tables

**Table 1:** Nutritional composition of meat, PBM burgers, and standard chow.

	Energetic Value kcal/100 g	Protein g/100 g	Fat g/100 g	Salt g/100 g	Carbohydrates g/100 g
Meat burger *	238	19	18	1	0.1
PBM burger *	231	16	16	1.7	5.8
Standard chow	87.45	18	1.5	-	-

* Nutritional composition declared on the label.

**Table 2:** Nutritional composition of improved chow.

	Components				
	Energetic Value kcal/100 g	Protein g/100 g	Fat g/100 g	Salt g/100 g	Carbohydrates g/100 g
Improved chow (with 23% lyophilized meat burger)	164.21	21.45	8.47	0.03	0.4
Improved chow (with 23% lyophilized PBM burger)	120	17.54	4.83	0.39	4.83
Standard feed	87.45	18	1.5	-	-

**Table 3:** Analytical methods and reagents for biochemical analysis.

Biochemical Parameter	Analytical Method	Reagent	CAT
AST	International Federation for Clinical Chemistry (IFCC) standardized kinetics using the pyridoxal phosphate method	Aspartate aminotransferase, Roche Diagnostics, GmbH, Mannheim, Germany (ASTL)	20764949322
ALT	International Federation for Clinical Chemistry (IFCC) standardized kinetics using the pyridoxal phosphate method	Alanine aminotransferase, Roche Diagnostics, GmbH, Mannheim, Germany (ALTL)	20764957322
CHOL	Spectrophotometric (enzymatic colorimetric) method	Cholesterol Roche Diagnostics, GmbH, Mannheim, Germany (CHOL2)	03039773190
HDL	Spectrophotometric (enzymatic colorimetric) method	HDL Cholesterol Roche Diagnostics, GmbH, Mannheim, Germany (HDLC4)	07528566190
LDL	Spectrophotometric (enzymatic colorimetric) method	LDL Cholesterol Roche Diagnostics, GmbH, Mannheim, Germany (LDL-C Gen.3)	07005717190
TG	Spectrophotometric (enzymatic colorimetric) method	Triglycerides Roche Diagnostics, GmbH, Mannheim, Germany (TRIGL)	20767107322
CR	Kinetic (enzymatic colorimetric) Jaffé method	Creatinine Jaffé, 2nd generation, Roche Diagnostics, GmbH, Mannheim, Germany (CREJ2)	04810716190
UR	Spectrophotometric (kinetic) method	Urea, Roche Diagnostics, GmbH, Mannheim, Germany (Ureal)	04460715190

**Table 4:** Weight gain and food intake of rats.

Parameters Mean (SD)	CF	MF	PF	CM	MM	PM
Initial weight (g)	258.83 (13.90)	237.50 (8.29)	279.33 (6.53)	447.83 (44.31)	452.33 (24.57)	433.50 (11.14)
Final weight (g)	276.01 (16.04)	250.66 (5.12)	293.04 (3.84)	489.83 (24.57)	482.67 (30.17)	485.17 (18.91)
Weight gain (g)	17.17 (18.77)	13.17 (10.81)	13.67 (11.79)	42.00 (30.98)	30.33 (28.31)	51.66 (24.83)
Mean food intake (g/day)	14.10 (3.23)	17.58 (7.75)	16.68 (5.91)	24.62 (5.71)	24.87 (5.55)	23.69 (3.52)
FER	1.21 (0.34)	0.74 (0.51)	0.81 (0.45)	1.70 (1.34)	1.21 (0.98)	2.18 (1.89)

**Table 5:** Serum values for leptin and leptin index.

Leptin (ng/mL)	CF	MF	PF	CM	MM	PM
Minimum	0.64	2.72	2.13	0.92	2.84	3.19
Maximum	2.34	4.01	3.62	3.02	7.25	5.85
Mean (SD)	1.25 (0.62)	3.40 (0.51)	2.81 (0.64)	1.82 (0.75)	4.86 (2.07)	4.37 (1.10)
Leptin index (ng/mL/g)	0.0045	0.0135	0.0096	0.0037	0.0100	0.0090

**Table 6:** Histopathological changes observed in the heart, liver, and kidney two months after daily administration of improved feed in three-month-old rats.

Histopathological Findings/Group	CM	MM	PM	CF	MF	PF
Liver						
Periportal inflammatory infiltrate/Multifocal lymphocytic infiltrates, mainly peri-portal (0 = absent, 1 = present)	0	1	1	1	0	1
Intraparenchymal inflammatory infiltrate/Multifocal lymphocytic infiltrates, diffuse in the liver parenchyma (0 = absent, 1 = present)	0	1	1	0	1	1
Microvacuolar dystrophy/Hepatic micro-vesicular steatosis, mainly centrilobular (0 = absent, 1 = present)	0/1	1	0	0/1	0	0/1
Portal fibrosis/portal fibrosis (0 = absent, 1 = reduced, 2 = moderate, 3 = well expressed)	0	0	0	0	0	0
Heart						
Mean right ventricular cord/thickness (mm)	1.16	0.83	1.16	0.76	0.66	0.83
Mean left ventricular cord/thickness (mm)	3.00	2.66	3.00	2.00	2.50	2.16
Subepicardial adipose tissue (0 = absent, 1 = present)	0	1	1	0	0	0
Intramyocardial adipose tissue (0 = absent, 1 = present)	0	1	0	0	1	1
Interstitial, subendocardial, perivascular fibrosis (0 = absent, 1 = present)	1	2	2	0	1	2
Ischemic lesions (0 = absent, 1 = present)	1	1	1	0	1	1
Kidney						
Kidney Interstitial inflammatory infiltrate (0 = absent, 1 = present)	0	0	0	0	0	0

## Data Availability

The original contributions presented in this study are included in the article. Further inquiries can be directed to the corresponding author.
